# Inflammasome Modulation by Chemotherapeutics in Malignant Mesothelioma

**DOI:** 10.1371/journal.pone.0145404

**Published:** 2015-12-21

**Authors:** Catherine Westbom, Joyce K. Thompson, Alan Leggett, Maximilian MacPherson, Stacie Beuschel, Harvey Pass, Pamela Vacek, Arti Shukla

**Affiliations:** 1 Department of Pathology and Laboratory Medicine, University of Vermont College of Medicine, Burlington, VT, United States of America; 2 Department of Medical Biostatistics, University of Vermont College of Medicine, Burlington, VT, United States of America; 3 Department of Cardiothoracic Surgery, New York University School of Medicine, New York, New York, United States of America; Virginia Tech University, UNITED STATES

## Abstract

Malignant mesothelioma (MM) is a fatal disease in dire need of therapy. The role of inflammasomes in cancer is not very well studied, however, literature supports both pro-and anti-tumorigenic effects of inflammasomes on cancer depending upon the type of cancer. Asbestos is a causative agent for MM and we have shown before that it causes inflammasome priming and activation in mesothelial cells. MM tumor cells/tissues showed decreased levels of inflammasome components like NLRP3 and caspase-1 as compared to human mesothelial cells or normal tissue counterpart of tumor. Based on our preliminary findings we hypothesized that treatment of MMs with chemotherapeutic drugs may elevate the levels of NLRP3 and caspase-1 resulting in increased cell death by pyroptosis while increasing the levels of IL-1β and other pro-inflammatory molecules. Therefore, a combined strategy of chemotherapeutic drug and IL-1R antagonist may play a beneficial role in MM therapy. To test our hypothesis we used two human MM tumor cell lines (Hmeso, H2373) and two chemotherapeutic drugs (doxorubicin, cisplatin). Through a series of experiments we showed that both chemotherapeutic drugs caused increases in NLRP3 levels, caspase-1 activation, pyroptosis and pro-inflammatory molecules released from MM cells. *In vivo* studies using SCID mice and Hmeso cells showed that tumors were smaller in combined treatment group of cisplatin and IL-1R antagonist (Anakinra) as compared to cisplatin alone or untreated control groups. Taken together our study suggests that chemotherapeutic drugs in combination with IL-1R antagonist may have a beneficial role in MM treatment.

## Introduction

Malignant mesothelioma (MM) is a neoplasm of pleural or peritoneal mesothelial cells with no successful therapy available. MM is caused by asbestos and is extremely resistant to chemotherapy and radiation [1]. Among other factors, a defect in apoptosis appears to be a major contributory factor in drug resistance. Inflammation also plays an important role in MM development as shown by our group as well as others [2, 3]. The inflammasome is a special component of the inflammation machinery that is comprised of NOD-like receptors (NLRs), a family of intracellular sensors [4]. NLRs like NLRP3 and others form a multiprotein complex with apoptotic speck-like protein containing a CARD domain (ASC) in response to stimuli, resulting in caspase-1 activation. Active caspase-1 can process pro-inflammatory cytokines like IL-1β and IL-18 into their mature form. IL-1β is well known to play a positive role in tumorigenesis of various cancers [5, 6] and has been implicated in proliferation of mesothelial cells, a cell of origin for MM [7]. Recently we have demonstrated that asbestos and erionite can prime and activate the NLRP3 inflammasome in human mesothelial cells and that an autocrine feedback loop for inflammasome regulation exists [8]. In the same study, treatment of MM tumor bearing SCID mice with IL-1R antagonist (Anakinra) resulted in significantly decreased levels of IL-8 and VEGF in peritoneal lavage fluid (PLF), suggesting that IL-1 cytokines may play a significant role in regulating other pro-tumorigenic cytokines and, therefore, tumorigenesis.

The role of inflammasomes in carcinogenesis is contrasting; they may inhibit tumor promotion by activating caspase-1 and cell killing, or may promote tumor growth by upregulating secretion of pro-inflammatory molecules like, IL-1β, IL-18, FGF2 and HMGB1. It has been reported that pro or anti tumorigenic effects of inflammasomes may depend on the cell type in which they are activated. For example, 2 groups independently reported that the absence of NLRP3 and caspase-1 attenuated chemical-induced colitis in mice [9, 10], whereas other groups have reported that different inflammasome knockout (KO) mice are more sensitive to chemical-induced colitis and tumorigenesis [11, 12]. Detailed studies by a later group demonstrated that NLRP3 expression by hematopoietic cells exerts anti-tumorigenic functions, whereas that from intestinal epithelial or stromal cells do not [11]. These studies suggest that inflammasomes from a different cell type of the same individual may have differing effects; thus; global KO mice may not be the appropriate tool to study the effect of inflammasomes.

For the present study, we hypothesize that NLRP3 inflammasome attenuation, which occurs in mesothelioma, enhances drug resistance, and hence there is a case for induction of inflammasome activation. The downside of this is more production of pro-inflammatory cytokines, especially IL-1β and related cytokines with a tumor growth promotion effect. Blocking IL-1β function would inhibit the latter effect. Treatment of MM cell lines with chemotherapeutic drugs resulted in priming and activation of NLRP3 inflammasome and increased secretion of pro-inflammatory molecules. Based on our preliminary findings we hypothesized that a combination of chemotherapeutic drug and IL-1 receptor antagonist (IL-1ra) may work better for MM treatment.

## Materials and Methods

### Cell culture and exposure to agents

Human peritoneal mesothelial LP9/TERT-1 (LP9) cells [13] were purchased from Brigham and Women’s Hospital, Harvard University, Boston, MA and grown as reported previously. Human MM cell lines, H2373, H2595, H2461, H2818, H2596 and HP-1 were contributed by Dr. Harvey Pass (New York University, New York, NY) [14]. Hmeso cells were isolated by Reale et al. [15]. Immortalized mesothelial cells (LP9) were similar to primary mesothelial cells in their responses to asbestos as previously published [8]. All cells were cultured as reported previously [16]. Cell lines were validated by STR DNA fingerprinting using the Promega CELL ID System (Promega, Madison, WI) as previously reported [17]. Human MM tumor tissues and normal counterparts were also obtained from Dr. Pass [18], without revealing their identity. Doxorubicin (Dox) was purchased from Sigma (St. Louis, MO) and cisplatin from Alfa Aesar (Ward Hill, MA). Dox and cisplatin concentrations for the present study were selected based on already published literature for MM [16, 19] or other cell lines [20].

### Western blot analysis

Western blot analysis was performed as described previously [21], using specific antibodies. Medium was collected, cells were lysed as previously described [22], and protein content in cell lysates was determined using the RC DC protein assay (Bio-Rad, Hercules, CA). Medium (500 μL) was concentrated using Amcion® ultracentrifugal filters with a 10 K membrane (Millipore, Billerica, MA) as described previously [8]. For some experiments, sample medium was concentrated using Strata Clean resin beads (Agilent Technologies, Santa Clara CA). Western blot analyses were performed as previously described [22] on both cell lysates (NLRP3, HMGB1) and concentrated supernatants (HMGB1, Capase1-p20, FGF2, IL-6). Rabbit polyclonal antibodies for HMGB1 (Abcam, Cambridge, MA), NLRP3 (Novus Biologicals, Littleton, CO) and Caspase-1-p20 (Cell Signaling, Danvers, MA) were used at 1:500 dilutions. QuantityOne was used to quantify band density. Beta-actin was used as a loading control for lysate samples. For conditioned medium and peritoneal lavage fluid (PLF) samples, equal loading was confirmed by Ponceau stain as no loading control is available (data not shown). Blots are representative of at least 2–3 different experiments.

### Quantitative real-time PCR (qRT-PCR)

Total RNA was prepared using an RNeasy plus mini kit according to the manufacturer’s protocol (Qiagen, Valencia, CA) as described previously [21]. Total RNA (1μg) was reverse-transcribed with random primers using the Promega AMV Reverse Transcriptase kit (Promega, Madison, WI) according to the recommendations of the manufacturer. Gene expression was quantified by TaqMan Real Time Q-PCR using the 7700 Sequence Prism Detector (Perkin Elmer Applied Biosystems, Foster City, CA) as described previously [23]. Duplicate or triplicate assays were performed with RNA samples isolated from at least 2 independent experiments.

### ELISA assay for IL-1β and IL-18

The Quantikine Human IL-1β/IL-1f2 Immunoassay (R&D Systems, Minneapolis, MN, measures predominantly mature IL-1β) was used on concentrated cell medium, prepared as described earlier, and the assay performed according to the manufacturer’s instructions as previously published [3]. IL-18 release was measured using the Human IL-18 ELISA kit (MBL International, Woburn, MA, measures predominantly active IL-18, 0.7% pro IL-18). Values were expressed as pg/mL of IL-1β or IL-18 from the original supernatant (non-concentrated).

### Caspase-1 activity assay

Caspase-1 activity was measured in cell lysates using the Caspase-1 Colorimetric Assay (R&D Systems, Minneapolis, MN), according to the manufacturer’s directions as previously reported [3]. In experiments where a colored compound like Dox was used, Caspase-Glo^®^ 1 Inflammasome Assay from Promega (Madison, WI) was used following the manufacturer’s instructions.

### Pyroptosis assessment

As caspase-1 activation is related to pyroptosis/apoptosis [8], we were interested in learning whether chemotherapeutic-induced MM cell death may in part be due to pyroptosis (caspase-1 dependent cell death) or not. For this purpose we pretreated MM cells with a specific caspase-1 inhibitor (40 μM Caspase-1 inhibitor VI (zYVADfmk), EMD Biosciences, Billerica, CA) for 1 h and subsequently with chemotherapeutic drugs, Dox (0–100 μM) or cisplatin (0–200 μM) for 24 h (Hmeso) and 72 h (H2373). Cell viability was then determined by MTS Assay CellTiter 96 Aqueous One Solution Cell Proliferation Assay (Promega) as per the manufacturer’s recommendations [16].

### Immunohisto/cytochemistry

Three MM tissue arrays were examined (these tissue arrays were obtained from Dr. Harvey Pass, NYU, NY, and were used before [24]. Each array contained 10–15 MM tumor sections from different patients, one lung carcinoma (on 2 of 3 arrays) and a section of normal lung. After deparaffinization and antigen retrieval [24] sections were blocked with 10% normal goat serum in 1X PBS for 1 h at RT. After washing in PBS, sections were incubated with 1:100 dilution of polyclonal anti rabbit NLRP3 (Enzo, Farmingdale, NY) overnight at 4°C in a humidified chamber. After washing in PBS, AlexaFluor® 647 anti-rabbit secondary antibody (Invitrogen, Grand Island, NY) was applied to sections for 1 h at RT in the dark. Each section was then washed in PBS again and treated with a 1:1000 dilution of Sytox® Green (Cell Signaling) for 10 minutes. For the negative control, one slide was stained as described above, but without primary antibody. MM cells from cultures were cytospun onto glass slides (50,000 cells/slide), fixed in 4% paraformaldehyde for 10 minutes and permeablized with 0.1% Triton-X for 10 minutes. The staining procedure described above was then performed starting with the 10% goat serum block. Confocal images were then taken with a Biorad MRC 1024ES Confocal Scanning Laser Microscope using Biorad LaserSharp imaging software (Advanced Imaging Concepts Inc.).

### *In vitro* and *in vivo* studies using the IL-1-ra, Anakinra

For *in vitro* experiments, cells were pre-treated with 100 ng/mL of IL-1ra (Insight Genomics, Falls Church, VA) for 1 h before administration of chemotherapeutic drugs [25]. For *in vivo* experiments, 2.25 X 10^6^ Hmeso MM cells in 500 μL sterile 0.9% NaCl (pH 7.4) were injected into the lower left quadrant of the peritoneal cavity of 8 week-old male Fox Chase SCID mice (5/group). One week post cell injection one group (n = 5) received cisplatin (2 mg/kg, ip, in saline) once a week for 2 weeks. The second group received 92 mg/kg Anakinra (Ana), ip (Kineret®) (Amgen, Thousand Oaks, CA) 2X daily for 3 weeks in 500 μL sterile 0.9% NaCl (pH 7.4) [26]. A third group received both cisplatin and Ana. Two control groups both received saline but at matching frequency with either cisplatin or Ana. The control groups were planned so as to study the effect of number of injections on tumor growth, if any. We did not observe any significant effect of number of injection on tumor growth; therefore the two groups were pooled for the purpose of analysis. Four weeks post-MM cell injections, mice were euthanized as described previously [17]. PLF was collected and animals were closely examined for the presence of tumors. Weights and volumes of tumors were determined as reported previously [2]. All experiments using mice were approved by the Institutional Animal Care and Use Committee (#12–004) at the University Of Vermont College Of Medicine.

### Determination of inflammatory cell profiles in PLF in the IP SCID mouse model

Following euthanization of mice, PLF was collected and processed for differential cell counts and cytokines as described previously [2].

### Analysis of cytokines in PLF

PLF was concentrated and analyzed for IL-1β, IL-18, IL-6, FGF2 and HMGB1 using ELISA or Western blot analysis protocols described above. In addition, a multiplex suspension protein array was performed using a custom designed Human Cytokine 16-plex panel (TNF α, IL-33, MCP-1, MMP-12, IL-1α, GM-CSF, G-CSF, IL-6, HGF, IL-8, VEGF, RANTES, RAGE, FGF basic, TFPI and BMP-2) magnetic Luminex screening assay (R&D Systems, Minneapolis, MN) following the manufacturer’s protocol as described previously [27]. Concentrations of each cytokine and chemokine were determined using Bio-Plex Manager Version 6.0 software (Bio-Rad, Hercules, CA). Data were expressed as pg cytokine/mL.

### Statistical analyses

In all *in vitro* assays, at least 2–3 independent samples were examined at each time point per group in duplicate or triplicate experiments. Data were evaluated by either ANOVA using the Student Neuman-Keul’s procedure for adjustment of multiple pairwise comparisons between treatment groups, the non-parametric Kruskal-Wallis and Mann-Whitney tests or a two-tailed Student’s t-test. Differences with p values ≤ 0.05 were considered statistically significant.

## Results

### MM cells and tumors show reduced inflammasome priming and activation

**Fig 1:**
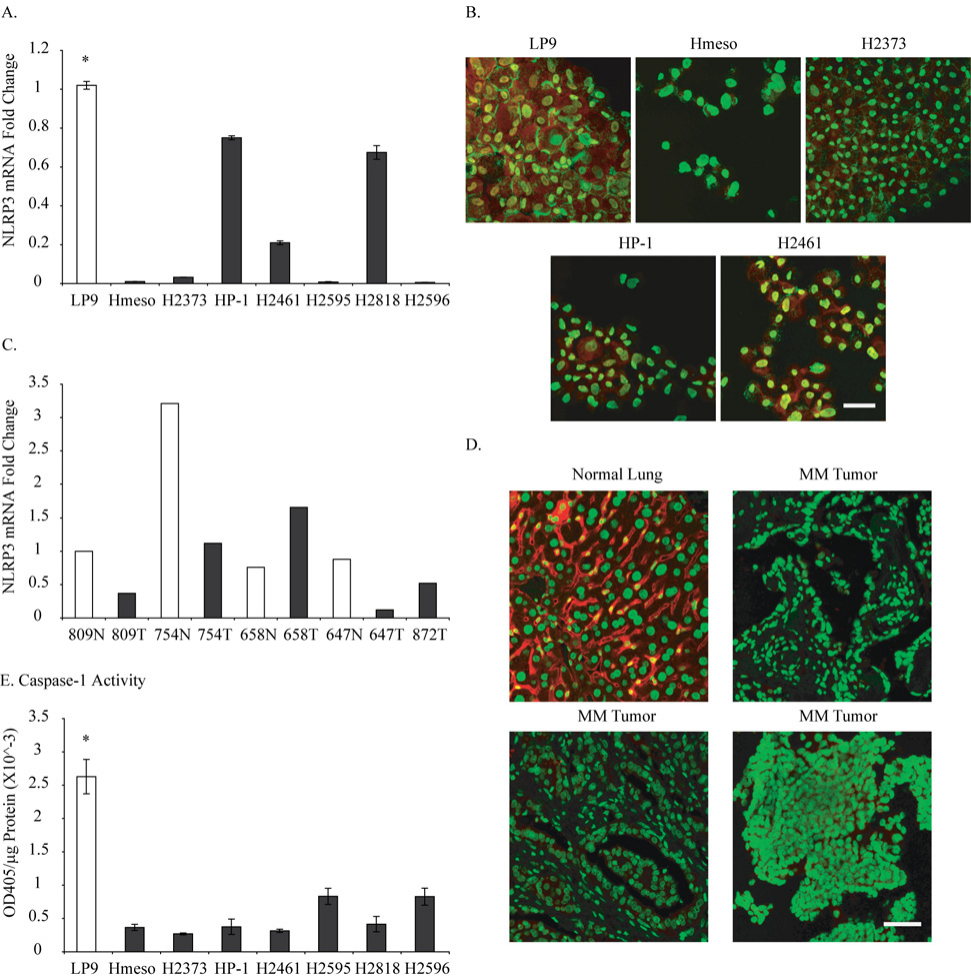
MM cells and tumors have low levels of NLRP3 protein and caspase-1 activity. A. Seven human MM cell lines were assessed for NLRP3 steady-state mRNA levels and compared with human mesothelial cell line (LP9), *p≤0.05 as compared to MM cell lines; B. NLRP3 protein levels in 4 human MM cell lines as compared to LP9 (red-NLRP3 protein, green-nucleus); C. NLRP3 mRNA levels in human MM tumor tissues (T) and normal counter parts (N); D. NLRP3 protein levels in human MM tumor tissue arrays as compared to normal lung (red-NLRP3 protein, green-nucleus); E. Caspase-1 activity in human MM cell lines as compared to LP9, *p≤0.05 as compared to MM cell lines. Scale bar = 50 microns.

Seven different MM tumor cell lines were assessed for NLRP3 mRNA levels relative to LP9. All MM cell lines showed lower levels of steady-state NLRP3 mRNA as compared to LP9 cells (Fig 1A). Selected MM lines were tested for protein levels of NLRP3 using immunocytochemistry and again these lines showed very low or non-existing levels of NLRP3 protein (Fig 1B, red-NLRP3 protein, green-nucleus) as compared to LP9 cells. RNA prepared from 4 different MM tumors (T) and normal counterpart (N) demonstrated low levels of NLRP3 in 3 out of 4 tumors (809T, 754T, 647T) as compared to their normal counterpart (Fig 1C). In addition, MM tumor tissue arrays stained for NLRP3 protein confirmed the results of MM tumors of low NLRP3 protein levels (Fig 1D, red-NLRP3 protein, green-nucleus). Caspase-1 activation, a marker of inflammasome activation, assessment in MM cells showed lower caspase-1 activity in these cells (Fig 1E). These results suggest a low level of inflammasome activity in MM cells and tumors.

### Chemotherapeutic drugs increase NLRP3 levels in MM cells

**Fig 2:**
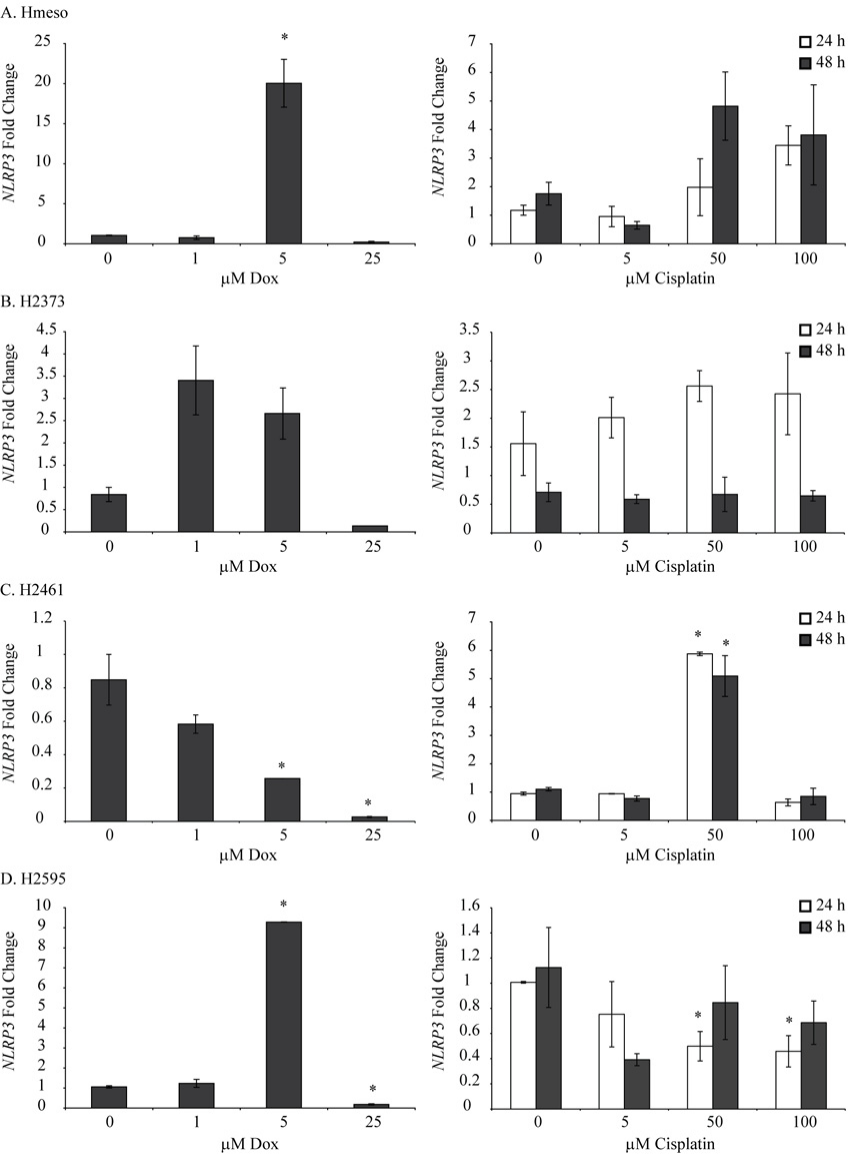
Chemotherapeutic drugs increase NLRP3 levels in human MM cell lines. Human MM cell lines were treated with different concentrations (0–100 μM) of cisplatin or Dox (0–25 μM,) for 24 or 48 h and steady-state NLRP3 mRNA levels were assessed by qRT-PCR. *p≤0.05 as compared to untreated (0) controls at the same time point.

**Fig 3:**
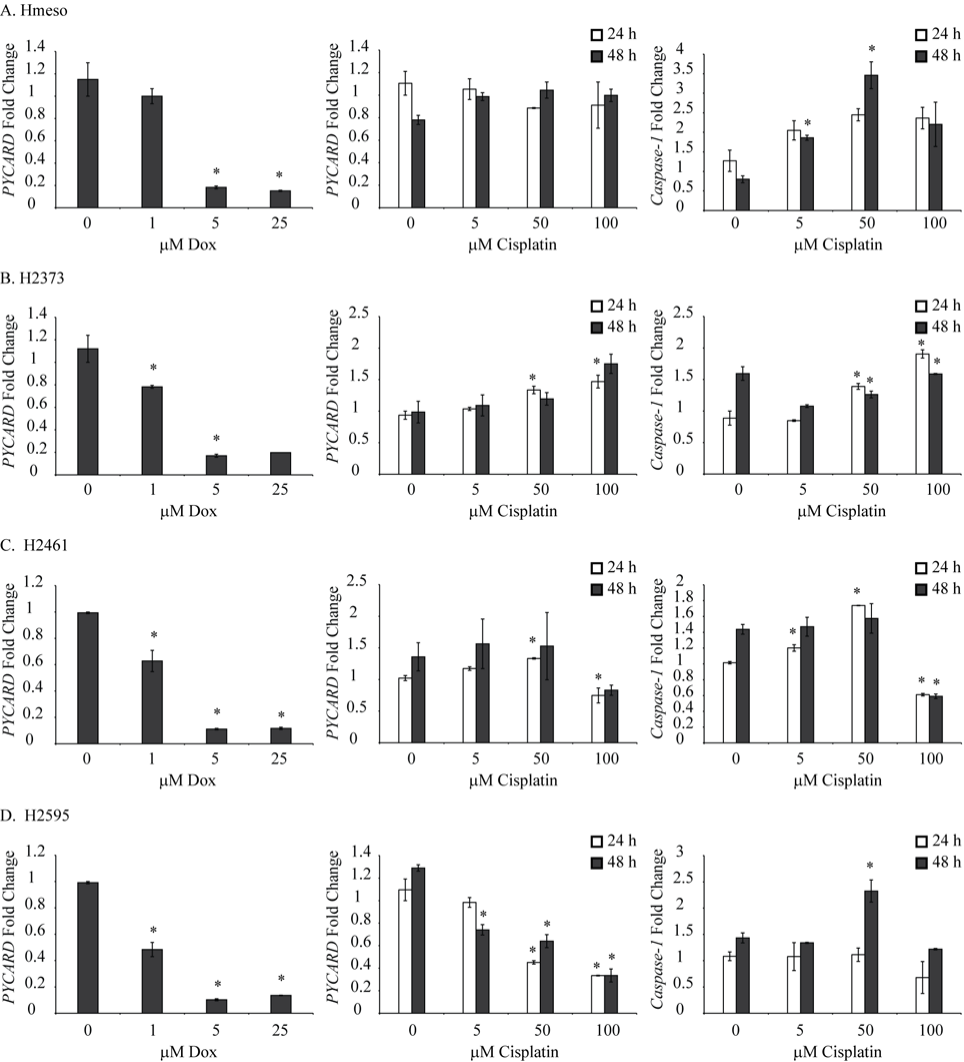
Chemotherapeutic drugs regulate PYCARD/ASC and pro-caspase levels in MM cell lines. Human MM cell lines were treated with different concentrations (0–100 μM) of cisplatin or Dox (0–25 μM) for 24 or 48 h and steady-state PYCARD/ASC or pro-caspase-1 mRNA levels were assessed by qRT-PCR. *p≤0.05 as compared to untreated (0) controls at the same time point.

To demonstrate the effect of chemotherapeutic drugs on the inflammasome status of MM cells, we selected 4 different MM cell lines and treated them with different concentrations of cisplatin or Dox for 24 h. Dox and cisplatin concentrations for the present study were selected based on already published literature for MM [16, 19] or other cell lines [20]. Viability of MM cells used in the present project, in response to different concentrations of Dox or cisplatin has been reported previously [19]. As shown in Fig 2 both drugs caused increases in the steady-state mRNA levels of NLRP3 in 3 out of 4 MM cell lines tested. Cisplatin was effective at higher doses in Hmeso cells (Fig 2A) and Dox was most effective at 5 μM concentration in all MM cell lines tested. For further studies we selected 100 μM cisplatin and 5 μM Dox. The lack of effect of Dox at higher concentration could possibly be due to cytotoxicity as reported previously [19]. In addition to NLRP3, we also measured steady-state mRNA levels of ASC/PYCARD and pro-caspase1 in MM cells with and without chemotherapeutic drugs and got varied results based on cell types and concentration of drugs used (Fig 3).

### Chemotherapeutic drugs prime and activate NLRP3 in Hmeso and H2373 cells

**Fig 4:**
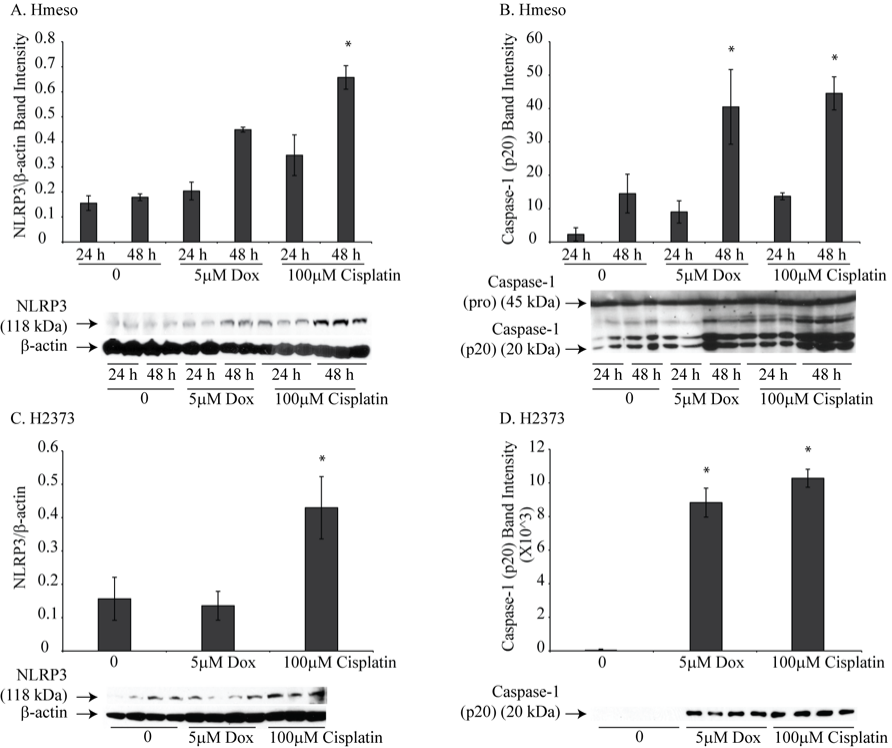
Chemotherapeutic drugs cause priming and activation of the NLRP3 inflammasome in MM cell lines. Treatment of Hmeso MM cells with Dox or cisplatin resulted in increased NLRP3 protein levels (A) and activation of inflammasome (B). Activation was measured by increased caspase-1 p20 release into the medium. *p≤0.05 as compared to untreated controls (0). (C) Cisplatin increased the protein levels of NLRP3 in H2373 MM cells and both Dox and cisplatin caused activation of caspase-1 in H2373 cells as measured by caspase-1 p20 release into the medium (D). *p≤0.05 as compared to untreated controls.

**Fig 5:**
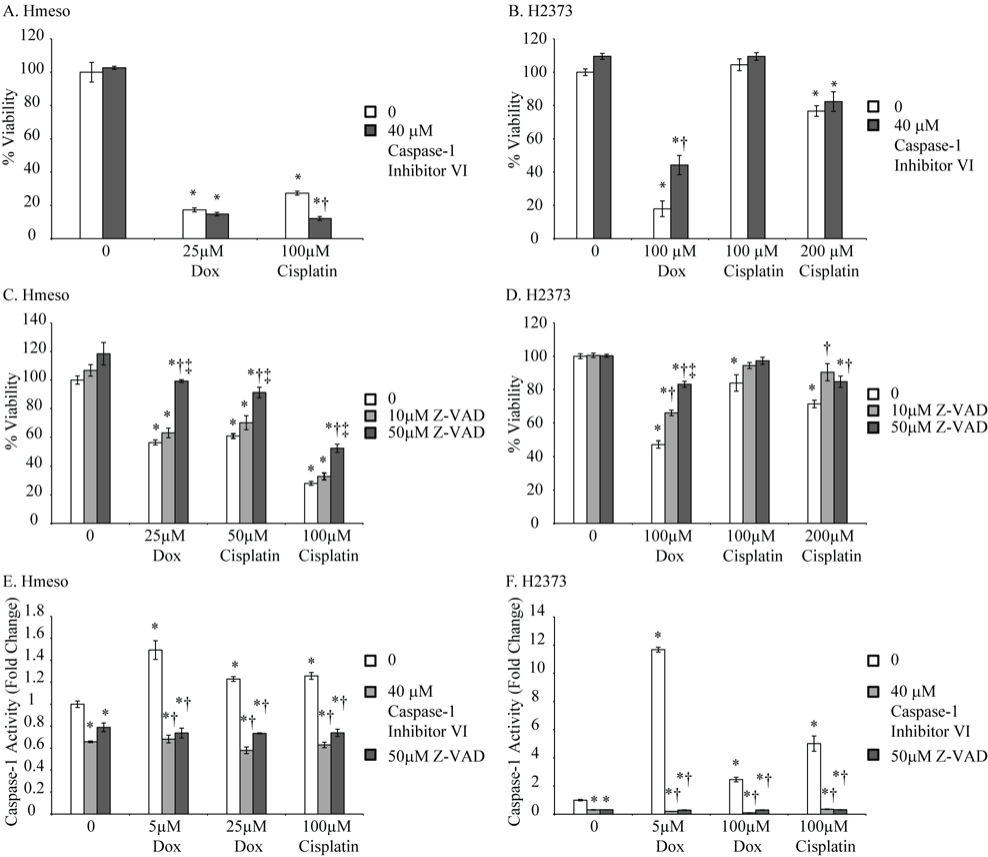
Chemotherapeutic drugs cause pyroptosis in MM cell lines. MTS assay performed to measure cell viability on Hmeso or H2373 MM cells in presence of Dox or cisplatin with and without specific caspase-1 inhibitor (A, B) or pan caspase inhibitor (C, D). The effect of these inhibitors on drug-induced caspase-1 activity was also performed in both MM cell lines (E, F). *p≤0.05 as compared to untreated controls (0); †p≤0.05 as compared to similar treatment without caspase- inhibitor, ‡ p≤0.05 as compared to low dose caspase inhibitor.

We selected two human MM cell lines, Hmeso and H2373, which form tumors in SCID mice, for further studies. Cell were treated with either Dox (5 μM) or cisplatin (100 μM) for 24 or 48 h and NLRP3 protein and caspase-1 activation was assessed. Drug concentrations used in this experiment were derived from results obtained from experiments in Fig 2. A significant increase in NLRP3 protein levels was observed with both drugs in Hmeso cells; however, only cisplatin caused a significant increase in NLRP3 levels in H2373 cells (Fig 4A and 4C). Caspase-1 activation, as measured by release of p20 subunit in the medium, was increased by both drugs in both MM cell types (Fig 4B and 4D). To show that activated caspase-1 contributes to drug-induced cell death (pyroptosis), we pretreated MM cells with a specific caspase-1 inhibitor (40 μM Caspase-1 inhibitor VI (zYVADfmk), EMD Biosciences, Billerica, CA) for 1 h and subsequently with chemotherapeutic drugs, Dox (0–100 μM) or cisplatin (0–200 μM) for 24 h (Hmeso) and 72 h (H2373). Higher concentrations of drugs were used for this experiment as low concentrations did not show any toxicity. Also, different concentrations of drugs were used for two different cell lines due to their differential susceptibility to drugs as reported previously (25). Cell viability was then determined by MTS Assay [16]. It is clear from Fig 5A, that Dox or cisplatin induced caspase-1 activation did not contribute significantly to Hmeso cell death, however, in H2373 cells caspase-1 inhibition resulted in a significant decrease in cell death by Dox (Fig 5B). With cisplatin, a similar trend of increased survival was also observed with caspase-1 inhibition in H2373 cells, but was not statistically significant. These data suggest that drug-induced killing is contributed in part by pyroptosis in some MM cells. Based on recent reports that there exists cross talk between the inflammasome and other caspases like caspase-8 [28], we hypothesized that inhibition of caspase-1 may cause a compensatory increase in caspase-8 activity, resulting in no effect of caspase-1 inhibition on cell viability as seen in Hmeso cells (Fig 5A). To test this we used a pan caspase inhibitor (z-vad-fmk, MP Biomedicals) on both MM cell lines. As shown in Fig 5C and 5D, pan caspase inhibitor significantly decreased drug-induced cell death in both cell types suggesting the possible compensation by other caspases in drug-induced toxicity. To demonstrate that these inhibitors are inhibiting caspase-1 activity, we performed caspase-1 activity assay in MM cells after treating them with chemotherapeutic drugs with or without caspase inhibitors. Results showed that both caspase inhibitors significantly attenuated drug-induced caspase-1 activity in both MM cell lines (Fig 5E and 5F).

### Chemotherapeutic drugs increase levels of pro-inflammatory cytokines in MM cells

**Fig 6:**
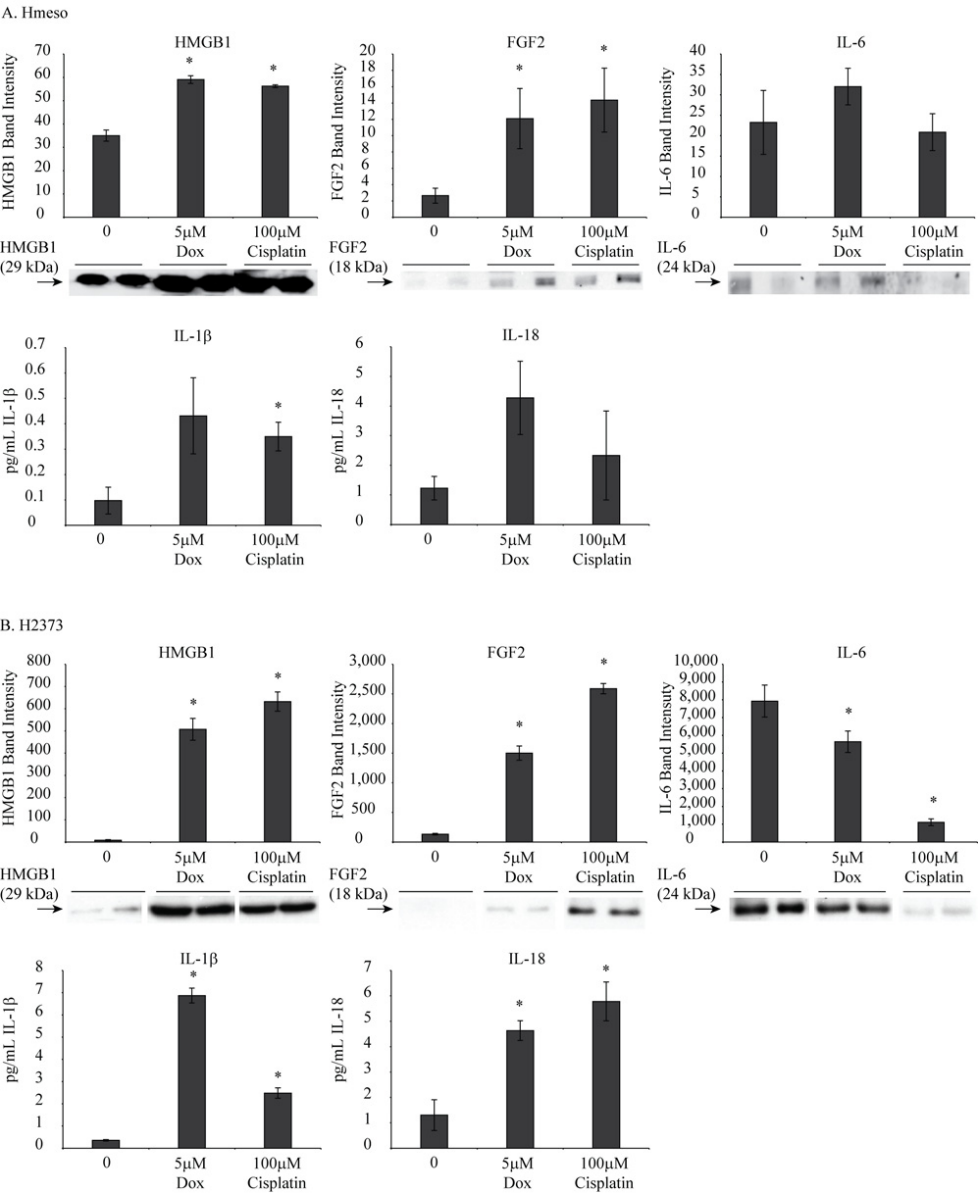
Chemotherapeutic drugs cause increased release of pro-inflammatory molecules from MM cells. (A) Hmeso MM cells treated with either Dox or cisplatin caused increased release of HMGB1, FGF2, IL-1β and IL-18 as measured by Western blot analysis or ELISA. No loading controls were included as analysis was performed in cell culture medium. (B) H2373 MM cells also showed increased release of various pro-inflammatory molecules in response to drugs. IL-6 levels were either decreased (H2373) or not significantly changed (Hmeso) by drug treatments. *p≤0.05 as compared to untreated controls (0).

We were also interested in understanding whether chemotherapeutics can cause increased inflammasome-related pro-inflammatory molecule release from MM cells. To execute this, Hmeso and H2373 cells were treated with Dox or cisplatin for 48 h. Cell lysates or conditioned medium were analyzed for various cytokines including IL-1β, IL-18, IL-6, FGF2, G-CSF and HMGB1. Fig 6 demonstrates that chemotherapeutic drugs caused increased levels of IL-1β, IL-18, FGF2 and HMGB1 in the culture medium from both cell types. IL-6 levels were either not changed significantly (Hmeso) or decreased (H2373) in response to drug treatments.

### IL-1β released did not show significant autocrine feed-back regulation of secreted cytokines

To show that IL-1β released can have an autocrine effect on inflammasome parameters, IL-receptor antagonist (IL-1Ra, Anakinra) was used. Hmeso cells were treated with chemotherapeutic drugs, Dox (5 μM) or cisplatin (100 μM), with or without Anakinra (100 ng/ml), and various inflammasome-related parameters were assessed. No significant effect of IL-1Ra at this concentration was observed at any of the parameters studied (data not shown) suggesting the lack of autocrine regulation in MM cells, unlike mesothelial cells reported previously [8].

### Combination of Anakinra and cisplatin had better effects on tumor reduction in *in vivo* xenograft model

**Fig 7:**
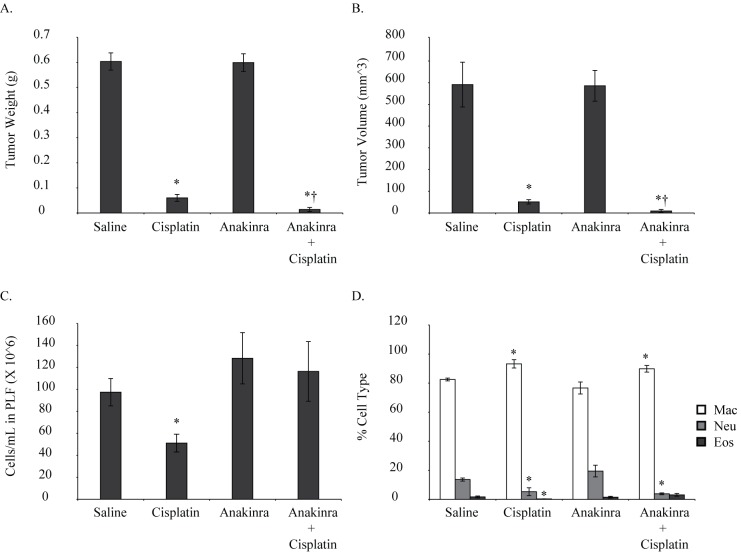
Combination of IL-1R antagonist and cisplatin attenuated MM tumor weight/volume in an intraperitoneal xenograft mouse model. SCID mice injected with Hmeso MM cells received either cisplatin (2mg/kg, ip, 1X/week for 2 weeks), Anakinra (92 mg/kg, ip, 2x daily for 3 weeks), Cisplatin and Anakinra together or saline. (A, B) tumor weights and volumes were drastically reduced in combination group. (C) Total cell count was not significantly affected by combination treatment. (D) Neutrophil counts were significantly lower in cisplatin and combination group. *p≤0.05 as compared to saline treated group; †p≤0.05 as compared to cisplatin alone (by Student’s‘t’ test).

Hmeso MM cells were injected ip into SCID mice and 1 week later cisplatin and/or Anakinra were injected ip as described earlier. Two daily doses of Anakinra were injected because half-life of Anakinra in mice is very short. Due to the short half-life of Anakinra in mice, a higher dose (100 mg/kg) is recommended for mice as opposed to the low dose (1 mg/kg) used in humans [26]. Anakinra at this dose is non-toxic except for being a local irritant in some cases due to low pH; however, no such effect was observed in our study. Four weeks post cell inoculation tumors were harvested and weighed. As this is an intraperitoneal model of MM, only one time point measurement of tumor and PLF is possible. PLF was collected and assessed for total and differential cell counts and cytokine levels (IL-1β, IL-18, IL-6, FGF2 and HMGB1). Two different control groups were planned so as to study the effect of number of injections on tumor growth, if any. We did not observe any significant effect of number of injections on tumor growth; therefore the two groups were pooled for the purpose of analysis. Weights of mice were measured before cell injection and every week thereafter till the end of the experiment. No significant weight loss was observed with Anakinra alone or Anakinra and cisplatin group as compared to untreated control at any time point (data not shown), suggesting no toxicity of the treatment. As shown in Fig 7A, cisplatin alone had a significant effect on tumor reduction, however, combination of cisplatin and Anakinra was more effective in tumor reduction than cisplatin alone († significantly different by ‘t’ test from cisplatin alone group). There was no significant effect of combination treatment on total cell counts (Fig 7C), whereas, neutrophil counts were significantly down in the cisplatin and combination group (Fig 7D). The number of neutrophils was lower in combination group as compared to cisplatin alone, however, did not reach significance. No histology or assays could be performed on tumor tissues from this experiment as combination group had almost no tumor (one out of 5 mice had a small tumor).

### Pro-inflammatory molecules in PLF were inhibited by Anakinra

**Fig 8:**
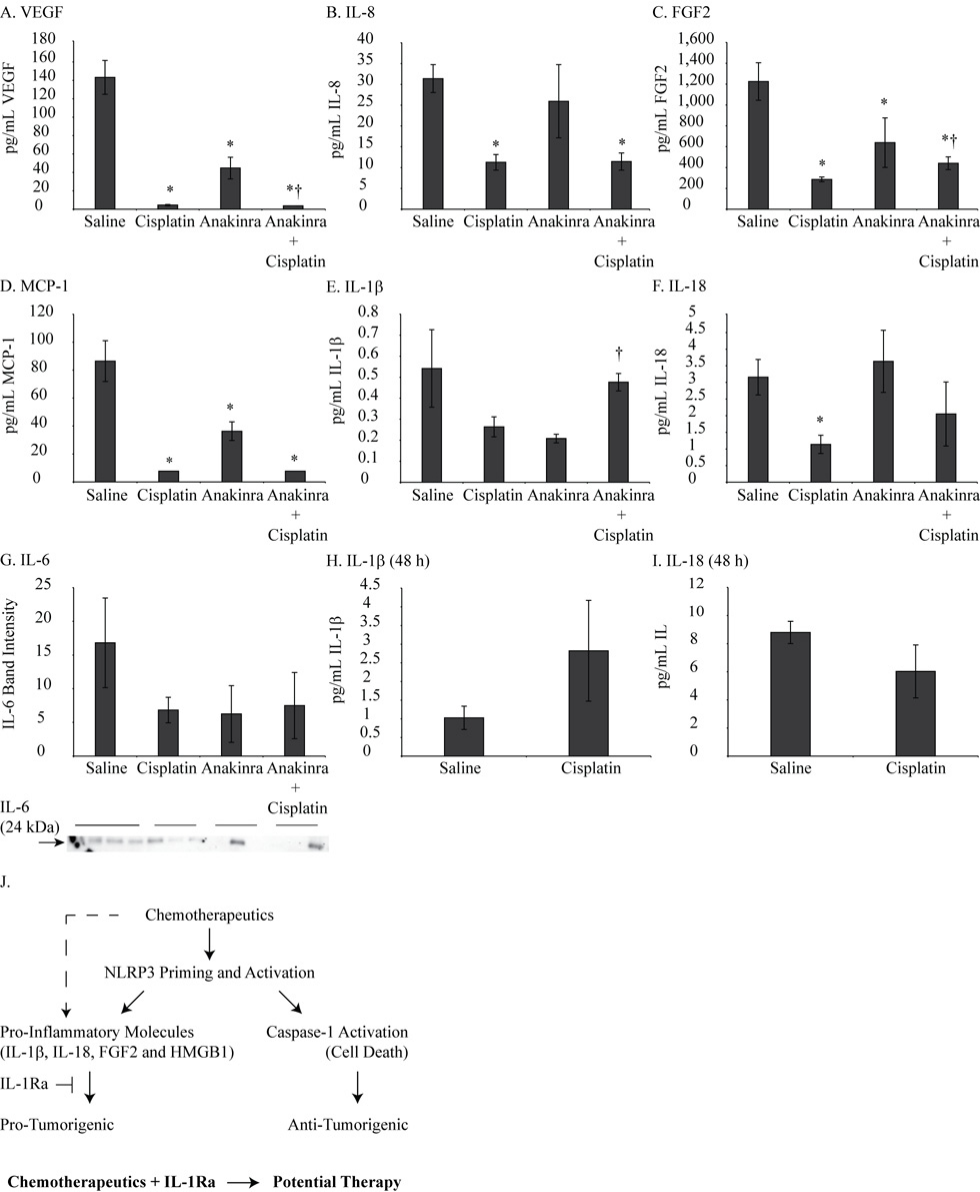
Combination of IL-1R antagonist and cisplatin reduced pro-inflammatory molecules in peritoneal lavage fluid of xenograft mouse model. (A-D) BioPlex performed on PLF showed significant reduction in FGF2, VEGF, MCP-1 and IL-8 in cisplatin alone or combination group. (E-G) A trend of decrease in IL-6, IL-1β and IL-18 was also observed by Western blot analysis and ELISA in PLF. *p≤0.05 as compared to saline; †p≤0.05 as compared to cisplatin alone. (H, I) ELISA detection of IL-1β and IL-18 levels in PLF of mice injected with Hmeso cells followed by cisplatin injection for 48h (J) A hypothetical schema showing how chemotherapeutic drugs in combination with IL-1 R antagonist could be a potential strategy for MM treatment.

Cytokine levels assessed in PLF by BioPlex (TNF α, IL-33, MCP-1, MMP-12, IL-1α, GM-CSF, G-CSF, IL-6, HGF, IL-8, VEGF, RANTES, RAGE, FGF basic, TFPI and BMP-2) showed decreased levels of MCP-1, FGF2, IL-8 and VEGF in cisplatin and combination group as compared to controls (Fig 8). IL-1β, IL-18 and IL-6 were also assessed in PLF by ELISA or Western blot analysis and results are presented in Fig 8. IL-1β levels were decreased by cisplatin as well as by Anakinra, however, levels were increased by combination treatment. These data show that chemotherapeutic drugs in combination with IL-1R antagonist may have potential in treating MM tumors (Summarized in Fig 8J schematic). As Anakinra is known to block IL-1 receptor, the ultimate effect could be the result of blockage of various cytokines regulated by IL-1 including IL-1α.

We observed increased secretion of IL-1β from MM cells *in vitro* in response to drugs (Fig 6) but a decreased amount in PLF in *in vivo* experiment (Fig 8). We hypothesize that it is due to differences in times, early vs. late. It has been reported earlier that in response to different stimuli IL-1β levels are initially increased. As IL-1β is known to regulate the synthesis of its own biological inhibitor IL-1r antagonist (IL-1ra) [29], the levels of IL-1β go down after initial increase as the positive feedback loop is interrupted. To test if IL-1β levels are increased at an earlier time point after cisplatin treatment in our *in vivo* system, we did a short term *in vivo* experiment, where SCID mice were injected with Hmeso cells and 1 week later were given single dose of cisplatin (2mg/kg, ip). Forty eight hours later, PLF was harvested and IL-1β and IL-18 levels were estimated by ELISA. In contrast to later time point results where we noticed decreased IL-1β levels (Fig 8E), we observed an increase in IL-1β levels (Fig 8H). Our findings suggest that there is an initial increase in IL-1β levels in response to chemotherapeutic drugs which later on subsides due to activation of feed- back regulatory mechanism. However, the initial increase could be sufficient to start signaling cascades for tumor promotion and blocking it by exogenous IL-1ra (Anakinra) before chemotherapy could be a better strategy for MM tumor treatment.

### NLRP3 regulation by chemotherapeutics in Hmeso MM cells

To understand the process of inflammasome regulation by chemotherapeutics in MM cells, Hmeso cells were treated with Dox alone or in combination with various inhibitors known to regulate inflammasomes in various systems (CA-074Me/cathepsin B inhibitor, cytochalasin D/actin polymerization inhibitor, KCl/blocks K^+^ efflux, KN-62/ATP receptor antagonist, NAC/antioxidant). A significant effect of cytochalasin D was observed in up-regulation of Dox-induced NLRP3, caspase-1 activation and HMGB1 secretion (Table 1). Cathepsin B inhibitor, CA-074Me, also resulted in a significant increase in Dox-induced caspase-1 and HMGB1; however, NLRP3 levels were down. NAC, KN-62 and KCl also had significant, but partial, effects on inflammasome components. We also observed further increase in NLRP3 levels and no change in caspase-1 and HMGB1 when Hmeso cells were treated with cathepsin B inhibitor before cisplatin treatment (Table 2). In contrast, ATP receptor antagonist further augmented the effect of cisplatin on all parameters of inflammasomes. Antioxidant NAC pretreatment mostly attenuated cisplatin-induced inflammasome activation (Table 2).

**Table 1:** NLRP3 regulation by Doxorubicin + inhibitors in Hmeso MM cells^†^.

Inhibitor	NLRP3	Caspase-1	HMGB1
CA-074Me (Cathepsin B)	↓*	↑***	↑**
Cytochalasin D (Actin Polymerization)	↑***	↑*	↑*
KCl (K+ Efflux)	↓	↑**	No change
KN-62 (ATP)	↓*	↑	No change
NAC (ROS)	↑	↓**	No change

†Upward arrow: increased; downward arrow: decreased; arrows with stars: significant changes; arrows without stars: changes did not reach significance; No change: no difference between groups.

*p≤0.05

**p≤0.01

***p≤0.001

**Table 2:** NLRP3 regulation by Cisplatin + inhibitors in Hmeso MM cells^†^.

Inhibitor	NLRP3	Caspase-1	HMGB1
CA-074Me (Cathepsin B)	↑*	No change	No change
KN-62 (ATP)	↑*	↑*	↑**
NAC (ROS)	↓	↑*, ↓**	↓

†Upward arrow: increased; downward arrow: decreased; arrows with stars: significant changes; arrows without stars: changes did not reach significance; No change: no difference between groups.

*p≤0.05

**p≤0.01

## Discussion

In this study we demonstrate that mesothelioma tumors/cells exhibit an attenuated NLRP3 inflammasome activation. Treatment with cisplatin or Dox enhances inflammasome expression and caspase-1 activity. Based on our hypothesis that this upregulation of the inflammasome has both an anti-tumor effect (through caspase- activation) and a tumor promoting effect (through enhanced cytokine production) we propose that combining chemotherapeutic drugs with IL-1 receptor antagonist may have a beneficial effect on MM tumor reduction.

The low caspase-1 activity could in part be responsible for the observed drug resistance in MM tumors, as active caspase-1 catalyzes activation of executioner caspase-7 and several other substrates resulting in apoptosis [30]. In support, it has been demonstrated that caspase-1 is down-regulated in human prostate cancers [31], and reintroduction of caspase-1 leads to greater sensitivity to radiation-induced killing *in vitro* [32]. Based on this report and our preliminary findings we hypothesized that chemotherapeutic drugs may activate inflammasomes and therefore, caspase-1, resulting in reduced drug resistance. As caspase-1 is involved in activating pro-inflammatory cytokines like IL-1β and IL-18, the negative effect will be increased levels of these pro-tumorigenic cytokines. To circumvent this, we proposed to use chemotherapeutic drugs in combination with IL-R antagonist (IL-1Ra, Anakinra) for MM tumor reduction.

First, using 2 human MM cell lines we demonstrated that chemotherapeutic drugs, Dox or cisplatin, can prime and activate NLRP3 as evident by caspase-1 activation. Although caspase-1 was activated in both MM cell lines, it impacted Dox-induced cell death in only H2373 cells. This lack of effect in Hmeso cells and by cisplatin in H2373 cells is possibly due to compensation by other caspases, as demonstrated by pan caspase inhibitor. In support recent literature demonstrates the cross talk of inflammasome with other caspases like caspase-8, in addition to caspase-1 [28, 33]. Furthermore, it has been reported that in dendritic cells Dox can release IL-1β by stimulation of non-canonical caspase-8 pathway [34]. In support of our findings other groups have also reported activation of inflammasome pathways by chemotherapeutic drugs in various cancer and non-cancer cells [35–37].

Inflammasome activation is also associated with release of mature pro-inflammatory cytokines like IL-1β, IL-18 and FGF2 (basic fibroblast growth factor). Our studies demonstrated that Dox or cisplatin treatment of MM cells produced significant amounts of these cytokines which are pro-tumorigenic molecules and favor tumor growth by various mechanisms. Anti-tumorigenic drugs producing pro-tumorigenic molecules by inflammasome activation have been reported by others also [38–40] and is a matter in need of further investigation. In addition, high mobility group box protein (HMGB1) another product of inflammasome activation was significantly upregulated by Dox or cisplatin in both MM cell lines. HMGB1 is a danger associated molecular pattern (DAMP) and can induce an inflammatory response. Chemotherapeutic drugs are also known to induce HMGB1 up-regulation and impart drug resistance by inducing autophagy in different cancers [41, 42]. Designing a combination treatment where we can keep the caspase-1 activation, but reduce the cytokine/HMGB effects could be a better approach. Therefore, we used IL-1R antagonist (Anakinra) in combination with chemotherapeutic drugs in an *in vitro* and *in vivo* system. Unlike our previous observation in mesothelial cells [8], no significant effects of IL-1R blocking were observed in MM cells suggesting that no autocrine feed-back regulation of inflammasome exists in MM cells.

Of the 2 MM cell lines used in most of the experiments, one is epithelioid (Hmeso) and another one is fibrosarcomatoid (H2373). The different effect could be attributed to the different nature of the two cell lines. Currently we have many epithelioid but only one fibrosarcomatoid MM cell line in stock, making the verification of this effect difficult.

An *in vivo* experiment was designed to confirm that chemotherapeutic drugs in combination with IL-1ra could be a better choice for inhibiting MM tumor growth. We injected SCID mice with human MM Hmeso cells and then treated them with cisplatin and/or Anakinra for 3 weeks. Cisplatin alone was very effective; however, cisplatin and Anakinra together had almost no tumors (in 4 out of 5 animals). Lack of tumor tissues in the combination treatment group prevented us from doing tumor tissue based analysis in this experiment. There was a significant decrease in the number of neutrophils in PLF of animals treated with the combination treatment, suggesting a contribution of inflammation in the process. BioPlex cytokine analysis performed on PLF showed a significant decrease in VEGF, IL-8, FGF2 and MCP1 in cisplatin alone and the combination group, as compared to the saline or Anakinra alone groups. VEGF, IL-8 and FGF2 are related to the process of angiogenesis and are directly or indirectly related to inflammasome activation and IL-1β release. IL-1β, IL-18 and IL-6 assessment in PLF also showed a decreasing trend but did not reach significance. Increased IL-1β levels observed in PLF in response to combination therapy could in part be due to increased IL-1ra in response to Anakinra as previously reported [8] thereby resulting in reduced IL-1β function but increased unbound IL-1β levels. There may be more reasons for this that deserves further attention. We have previously reported attenuated levels of MCP1 and VEGF by Dox in PLF of MM tumor bearing SCID mice, however, IL-6 and IL-8 levels were significantly increased [3]. This study has limitation of using immunocompromised mice as we don’t know the contribution of adaptive immune cells in the process of inflammasome activation or IL-1β release in response to chemotherapeutic drugs. To resolve this, further studies are in progress using allograft mouse models that will also educate us about the effect of host response in chemotherapy. These are very encouraging data suggesting that manipulation of inflammasome may work to control MM tumor growth by regulating various aspects of tumorigenesis. Currently we are in the course of studying this process in detail, with a lower (single) dose of cisplatin alone and in combination with pemetrexed, and with a larger number of animals /group, so as to understand the underlying mechanisms.

Taken together our study is first to suggest that chemotherapeutic drugs can activate NLRP3 inflammasomes in MM tumors resulting in caspase activation and increased cell death. Activation of the inflammasome is also accompanied by increased secretion of pro-inflammatory cytokines. Thus, a combination of chemotherapeutic drugs and IL-1R antagonist could be a possible strategy for inhibiting MM tumor growth. Our report is a very small first step in this direction and detailed *in vivo* studies with various models, chemotherapeutic drugs and tools to antagonize IL-1β are required to confirm the findings of this study and are presently in process.
